# TMUB1 Correlated with Immune Infiltration Is a Prognostic Marker for Colorectal Cancer

**DOI:** 10.1155/2022/5884531

**Published:** 2022-07-26

**Authors:** Tao Hao, Hanqiang Yu, Daqi Huang, Qian Liu

**Affiliations:** ^1^Department of General Surgery, The First Affiliated Hospital of Jinan University, Guangzhou 510632, China; ^2^Department of Radiology, The Affiliated Hospital of Binzhou Medical College, Binzhou, 256600 Shandong Province, China; ^3^Department of Cardiology, The Affiliated Hospital of Binzhou Medical College, Binzhou, 256600 Shandong Province, China; ^4^Laboratory of Molecular Cardiology, The First Affiliated Hospital of Shantou University Medical College, Shantou, China

## Abstract

**Background:**

Transmembrane and ubiquitin-like domain-containing protein 1 (TMUB1) is overexpressed in a large number of liver and esophageal tumors. However, only a few reports on the clinical significance of TMUB1 in colorectal cancer (CRC) exist.

**Methods:**

Here, we evaluated the clinical significance and potential biological role of TMUB1 using bioinformatics analysis. Univariate and multivariate analyses were performed to evaluate the relationship of TMUB1 with clinicopathological features. Gene set enrichment analysis (GSEA) was performed to identify the biological function of TMUB1, while any associations between the expression of TMUB1 and the infiltration of 24 immune cells were analyzed using simple-sample GSEA.

**Results:**

TMUB1 was significantly overexpressed in CRC tissues compared with normal controls. The high expression of TMUB1 in CRC was associated with T stage, neotype, and residual tumor. Moreover, TMUB1 was identified as an independent factor of poor disease-free survival (DFS) and short overall survival (OS). GSEA demonstrated that TMUB1 was related to hypoxia, angiogenesis, adipogenesis, inflammatory response, IL6-JAK-STAT3 signaling, apoptosis, mitotic spindle, and IL2-STAT5 signaling. The expression of TMUB1 negatively correlated with the abundance of T helper cells, Tcm cells, macrophages, and Th2 cells, whereas it positively correlated with the abundance of several immune cell types, including CD56bright and CD56dim NK cells.

**Conclusions:**

The high expression of TMUB1 is closely related to a poor prognosis in patients with CRC. TMUB1 may be a potential prognostic biomarker and be used for therapeutic approaches in CRC.

## 1. Introduction

Colorectal cancer (CRC) is the third most common type of cancer and the fourth most deadly cancer worldwide [1]. The incidence of colorectal cancer has been on a rise in patients younger than 50 years old, with many patients being diagnosed at the advanced stages of the disease, thereby losing the opportunity for surgery. Early detection, interventions, and improved treatments are important to reduce morbidity and mortality associated with CRC [2]. The pathogenic mechanisms underlying CRC include chromosomal instability (CIN), microsatellite instability (MSI), and the CpG island methylator phenotype (CIMP) [3]. These alterations can be measured and used as biomarkers. Although some biomarkers are used for diagnosis, consistent and sensitivity biomarkers are still lacking [4]. Hence, searching for new, specific, and sensitive molecule biomarkers is required to improve prediction, diagnosis, prognosis, and therapy in CRC.

Abnormal proliferation may occur may cause tumorigenesis. Transmembrane and ubiquitin-like domain-containing protein 1 (TMUB1), first reported in 2005, is significantly increased during liver regeneration [5]. TMUB1 is overexpressed in a high number of human tumors. The overexpression of TMUB1 in tumor cell lines was shown to strongly reduce proliferation by arresting the cell cycle in G0/G1 [6]. TMUB1 is a ubiquitin-like protein shuttling between the cytoplasm and the nucleus. Many investigations showed that TMUB1 not only controls proliferation but also plays an important role in apoptosis, cycle regulation, and genomic stability [7]. However, the expression of TMUB1 and its potential prognostic impact on colorectal cancer (CRC) has not yet been explored.

In this study, we evaluated the differential expression of TMUB1 between specimens from patients with CRC and normal tissues, performed correlation analysis between the TMUB1 expression and clinicopathological factors to assess the prognostic role of TMUB1, and identified relevant pathways and immune cell types associated with the high level of expression of TMUB1 observed in tumor samples from patients with colorectal cancer.

## 2. Materials and Methods

### 2.1. RNA-Sequencing Data and Clinic Information Analysis from TCGA Data

Gene expression data (level 3 HTSeq-FPKM) and clinical information were collected from a total of 619 cases of CRC and rectal cancer from TCGA ((https://portal.gdc.cancer.gov/), including 50 cases with paired paracancer samples). Exclusion criteria included samples without clinical information. The HTSeq-FPKM were converted to TPM (transcripts per million reads) for subsequent analysis. According to median value of the TMUB1 expression, tumor samples were divided into high- and low-expression groups. All data used in the study were in accordance with publication guidelines stated by TCGA (http://cancergenome.nih.gov/publicaionguidelines). Characteristics of patients, including gender, race, TNM stage, and tumor location, were recorded and are listed in Table 1. The expression of TMUB1 in paired tumor and adjacent samples and nonpaired samples was analyzed using the Wilcoxon signed rank test and Wilcoxon rank sum test, respectively.

### 2.2. Survival Analysis

The Kaplan-Meier (KM) Plotter (http://kmplot.com) was used to calculate the overall survival (OS) and disease-free survival (DFS) to analyze the prognosis of patients with tumors. Univariate and multivariate Cox regression analyses were used to compare the prognostic value of the TMUB1 expression and other clinical features as definite factors.

### 2.3. Gene Set Enrichment Analysis

We performed GSEA using the *R* package clusterProfiler (3.8.0) [8] to predict differential pathways and significant functions between high- and low-expression groups. Each analysis was based on 1,000-times gene set permutations. The expression level of TMUB1 was used as a phenotype label. The pathways enriched in each phenotype were analyzed based on the adjusted *P* value of less than 0.05, FDR *q* value of less than 0.25, and normalized enrichment score of more than 1 in the enrichment of the MSigDB Collection (h.all.v7.0.symbols.gmt [Hallmarks]).

### 2.4. Immune Cell Characteristics Analyzed by ssGSEA

Simple-sample GSEA (ssGSEA) was performed using the GSVA package [9] in *R* (3.6.3) to analyze the infiltration of 24 types of tumor-infiltrating immune cells in CRC tissue samples. We quantified the relative score of each immunocyte from the gene expression profile of each sample. Wilcoxon rank sum test was performed to analyze the infiltration of immune cells between low- and high-expression TMUB1 groups, while Spearman correlation was used to analyze the correlation between TMUB1 expression and these immune cells.

## 3. Results

### 3.1. TMUB1 Overexpression in Patients with Colorectal Cancer

**Figure 1:**
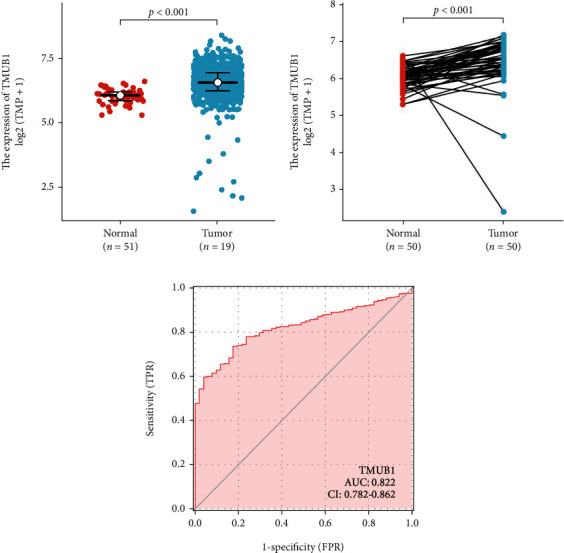
(a) Expression level of TMUB1 in tumor and adjacent normal samples analyzed using the Wilcoxon rank sum test. (b) Expression level of TMUB1 in paired tumor and normal samples analyzed using the Wilcoxon signed rank test. (c) Receiver operating characteristic (ROC) curves of the expression of TMUB1 in the TCGA. FPR: false positive rate; TPR: true positive rate.

Analysis using the Wilcoxon rank sum test revealed that the expression level of TMUB1 in 619 tumor tissues was higher than that in 51 normal tissues (*P* < 0.001, Figure 1(a)). Likewise, using the Wilcoxon signed rank test, we observed that the expression of TMUB1 was significantly higher in 50 tumor tissues than that in the 50 paired normal tissues in the TCGA cohort (*P* < 0.001, Figure 1(b)). Both results showed the significant overexpression of TMUB1 in patients with CRC. We further noticed that the ROC curves of the TMUB1 expression showed that the status of the TMUB1 expression could be served as a biomarker for CRC, (AUC: 0.822) in the TCGA dataset (Figure 1(c)).

### 3.2. Association between TMUB1 Expression and Clinicopathological Variables

**Figure 2:**
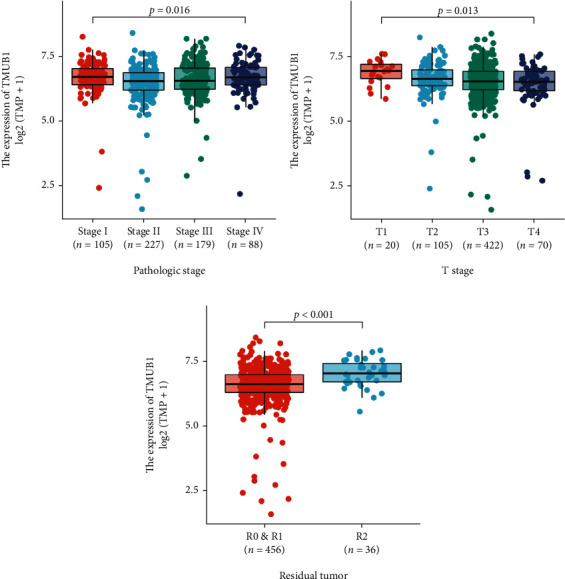
Association between the expression level of TMUB1 and clinicopathological characteristics in patients with colorectal cancer. (a) Pathologic stage. (b) T stage. (c) Residual tumor.

We detected a significant difference between TMUB1 overexpression and T stage (*P* < 0.001), pathologic stage, and residual tumor when the Kruskal-Wallis rank sum test was used (Figure 2). The high TMUB1 expression was significantly correlated with T stage (*P* = 0.046) and residual tumor (*P* = 0.001) using chi-square test or Fisher's exact test (Table 2). We also analyzed the relationship between clinicopathological features of CRC and TMUB1 TPM values using the logistics regression method and found that TMUB1 significantly correlated with T stage (OR = 0.63, 95% CI: 0.42-0.94, *P* = 0.017) and residual tumor (OR = 2.35, 95% CI: 1.20-4.88, *P* = 0.016) (Table 3).

### 3.3. High TMUB1 Expression Associated with Adverse Outcomes in Colorectal Cancer

**Figure 3:**
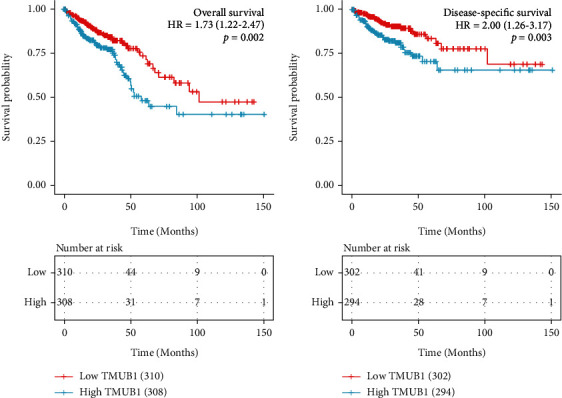
Impact of the TMUB1 expression on overall survival and disease-specific survival in patients with colorectal cancer.

KM analysis revealed that the TMUB1 overexpression significantly correlated with shorter OS (HR = 1.73, 95% CI: 1.22-2.47, *P* = 0.002) and poorer disease-specific survival (DFS) (HR: 2.00, 95% CI: 1.26-3.17, *P* = 0.003) (Figure 3).

More specifically, univariate analysis revealed that the TMUB1 expression (HR: 1.73, 95% CI: 1.216-2.473, *P* = 0.002) significantly associated with a poor OS. We found that other clinicopathological variables associated with poor OS included T stage, N stage, M stage, pathologic stage, age, residual tumor, and CEA level. Using multivariate analysis, we observed that TMUB1 (HR: 2.077, 95% CI: 1.101-3.921, *P* = 0.024) remained independently associated with OS along with pathologic stage (HR: 7.127, 95% CI: 1.529-33.227, *P* = 0.012) and age (HR: 2.886, 95% CI: 1.389-5.996, *P* = 0.005) (Table 4).

Similarly, univariate analysis revealed that the TMUB1 expression (HR: 2.003, 95% CI: 1.264-3.174, *P* = 0.003) was significantly associated with a poor DFS. We also observed that other clinicopathological variables associated with poor DFS included T stage, N stage, M stage, race, residual tumor CEA level, and pathologic stage. Using multivariate analysis, we found that the TMUB1 expression (HR: 2.538, 95% CI: 1.204-5.350, *P* = 0.014) remained independently associated with DFS along with M stage (HR: 4.5402.538, 95% CI: 1.574-13.096, *P* = 0.005) (Table 5).

### 3.4. GSEA Identified TMUB1-Related Signaling Pathways

**Figure 4:**
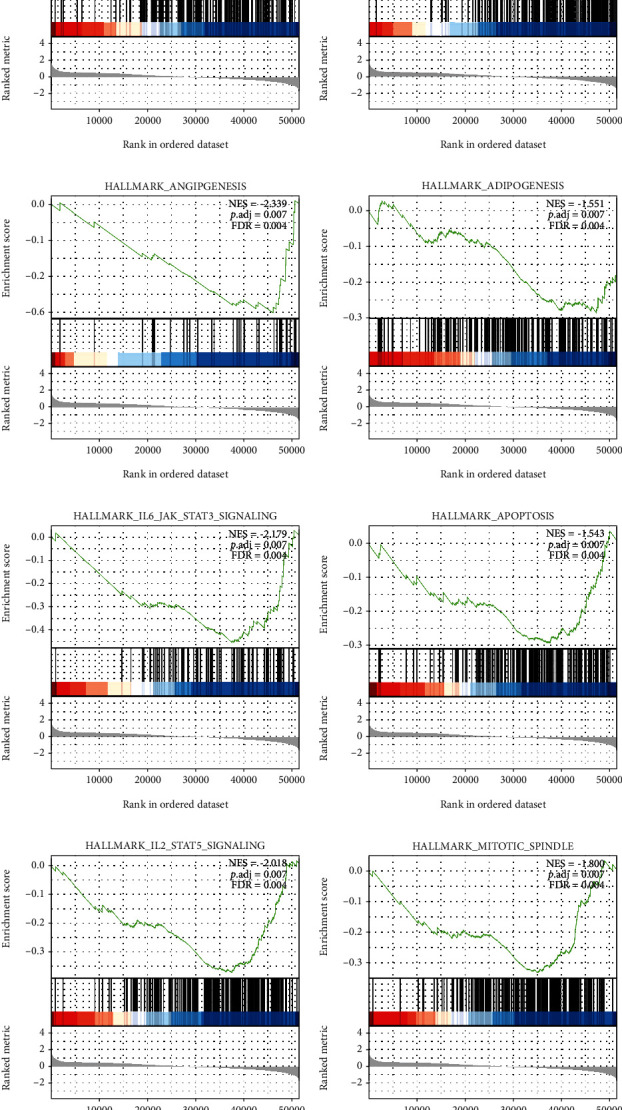
Enrichment plot from GSEA showing several pathways differentially enriched in TMUB1-related colorectal cancer, including hypoxia, inflammatory response, angiogenesis, adipogenesis, IL6-JAK-STAT3 signaling, apoptosis, mitotic spindle, and IL2-STAT5 signaling. ES: enrichment score; NES: normalized ES; adj. *P* val: adjusted *P* value.

We conducted GSEA between the datasets of low and high TMUB1 expression to identify differentially activated signaling pathways in CRC (Figure 4). Accordingly, GSEA showed that many key signaling pathways, such as hypoxia, inflammatory response, angiogenesis, adipogenesis, IL6-JAK-STAT3 signaling, apoptosis, mitotic spindle, and IL2-STAT5 signaling, were differentially enriched in the TMUB1 high-expression phenotype.

### 3.5. Association between TMUB1 Expression and Immune Infiltration

**Figure 5:**
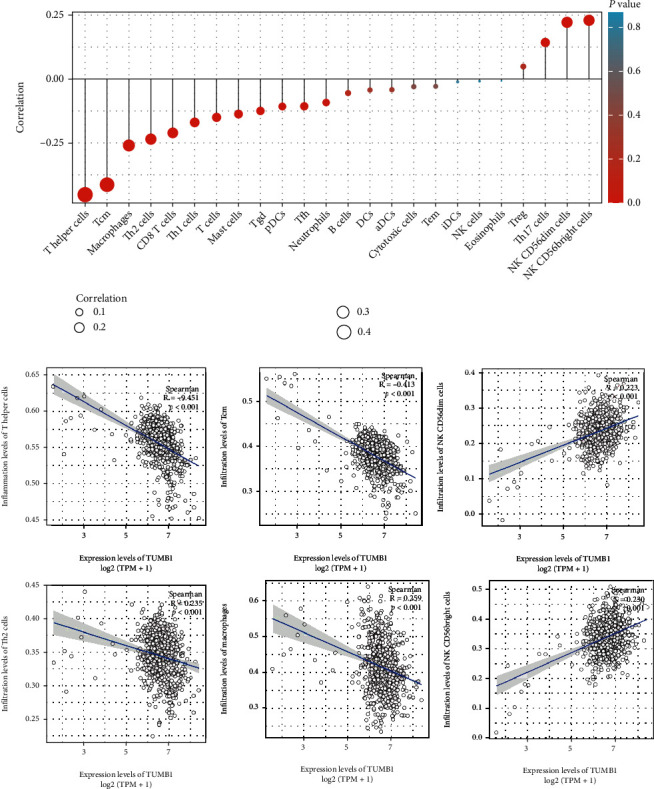
(a) Expression level of TMUB1 associated with immune infiltration in the tumor microenvironment. The size of dots represents the absolute value of Spearman's *r*. (b) Correlation between the relative enrichment score of the expression level of TMUB1 and cells.

Using the ssGSEA, we found that the TMUB1 expression negatively correlated with the abundance of several immune cell types, including T helper cells (*r* = −0.451, *P* < 0.001), Tcm cells (*r* = −0.413, *P* < 0.001), macrophages (*r* = −0.250, *P* < 0.001), and Th2 cells (*r* = −0.234, *P* < 0.001), whereas it positively correlated with the abundance of several immune cell types, including CD56bright (*r* = −0.230, *P* < 0.001) and CD56dim NK cells (*r* = −0.223, *P* < 0.001) (Figure 5).

## 4. Discussion

TMUB1, as a ubiquitin-like transmembrane protein shuttling from nucleus to cytoplasm, plays a significant role in controlling proliferation and genomic stability [6]. Moreover, TMUB1 is overexpressed in a large number of CNS, liver, and esophageal tumors. To the best of our knowledge, the TMUB1 expression, and its potential prognostic impact on CRC, has not yet been explored. Thus, the potential role of TMUB1 in CRC was the focus of the present study.

Bioinformatic analysis of high throughput RNA-sequencing data from TCGA demonstrated that the TMUB1 overexpression in CRC was associated with T stage, residual tumor, and poor prognosis, suggesting that TMUB1 may serve as a prognostic marker in CRC.

However, TMUB1 was demonstrated to be negatively correlated with HCC pathological malignancy [10] as low expression of TMUB1 correlated with poor prognosis in patients with HCC. This discrepancy may be attributed to TMUB1 playing different roles in different tissues. To this end, any TMUB1-specific functions in specific tissues should be explored.

Enrichment analysis of target gene sets using GSEA revealed some important networks of transcription factors and target kinases. More specifically, using GSEA, we observed that the TMUB1 overexpression was associated with hypoxia, inflammatory response, angiogenesis, adipogenesis, IL6-JAK-STAT3 signaling, apoptosis, mitotic spindle, and IL2-STAT5 signaling. Collectively, these results suggested that TMUB1 may play a key role in the molecular mechanism underlying CRC tumorigenesis. Further, these findings were consistent with the fact that hypoxia and inflammation are typical characteristics of cancer [11]. The vasculature is an important microenvironmental component and a potential therapeutic target of CRC [12]. Abdominal visceral fat is a well-recognized risk factor for CRC and fat as a dietary risk factor has also been associated with an increased risk for CRC [13]. In priming liver regeneration, the increased level of interleukin 6 (IL-6) was shown to upregulate TMUB1 expression [14, 15], with TMUB1 regulating hepatocyte proliferation via STAT3 pathway [16]. Moreover, TMUB1 exerts a regulator effect in stabilizing p53 and directing p53 mitochondrial apoptosis and cytoplasmic localization [17, 18]. TMUB1 is a main regulator of protein stability in the cell cycle. For instance, during the S phase of centrosome duplication, TMUB1 localized in the gap between centrosomes. The presence of two centrosomes during mitosis is critical for the formation of the bipolar mitotic spindles. Accordingly, knocking down TMUB1 led to abnormal spindle formation in cells, with TMUB1-silenced cells showing a high degree of centrosome amplification during mitosis, associated with multinucleated cells and multipolar spindles [18]. However, the association between the TMUB1 expression and IL2-STAT5 signaling was first the first time, and the regulatory mechanism underlying this association needs to be further elucidated.

Tumor cell-intrinsic factors shaping the tumor immune microenvironment underlie heterogeneity of immune cell infiltration and influence the outcome of immunotherapy [19]. In the tumor microenvironment, cancer cells and immune cells exert their effects by either promoting or repressing anticancer immunity. For instance, densities of T follicular helper (Tfh) cells increase along with tumor progression [9]. Tumor-infiltrating lymphocytes including NK cells and macrophages, T cells, secrete various factors which affect the microenvironment outside the tumor and closely related to the progression and prognosis of CRC.

In our study, the TMUB1 expression negatively correlated with the abundance of T helper cells, Tcm cells, macrophages, and Th2 cells, whereas it is positively correlated with the abundance of several immune cell types, including CD56bright and CD56dim NK cells. Hence, the correlation between immune cells and TMUB1 suggested that TMUB1 plays a complex role in regulating cancer immunity because of the various roles it plays in immune cells. There were significant differences in the infiltration levels of certain types of immune cells between the two groups, which may indicate that TMUB1 can affect the prognosis of CRC by affecting the level of immune infiltration.

However, there are some limitations in the study. The correlation between TMUB1 and CRC should be verified at cellular and organismal levels. Further studies with a large sample size and prospective design are warranted to avoid the selective bias and recall bias and achieve more meaningful outcomes. As the prediction of protein expression using mRNA expression levels was far from perfect, the correlation between the mRNA and protein expression of TMUB1 is required in further studies. In addition, wet lab work on the mechanisms underlying the TMUB1 functions is required to avoid missing any important signaling pathways explaining the mechanism of TMUB1 function in CRC.

## 5. Conclusions

In conclusion, the TMUB1 expression may be a potential prognostic molecular marker of poor survival in CRC. Moreover, IL6-JAK-STAT3 signaling, apoptosis, angiogenesis, adipogenesis, IL2-STAT5 signaling, inflammatory response, and TNFA signaling via NF-KB may be among the key pathways regulated by TMUB1 in CRC. TMUB1 may perform an immune regulation function in CRC. Further experimental validation is required to exhibit verify the biological impact of TMUB1.

## Figures and Tables

**Table 1:** Characteristics of patients with colorectal cancer based on TCGA.

Characters	Level	Overall
*n*		619
T stage (%)	T1	20 (3.2%)
	T2	105 (17.0%)
	T3	422 (68.2%)
	T4	70 (11.3%)
N stage (%)	N0	351 (57.0%)
	N1	150 (24.4%)
	N2	115 (18.7%)
M stage (%)	M0	459 (84.1%)
	M1	87 (15.9%)
Pathologic stage (%)	Stage I	105 (17.5%)
	Stage II	227 (37.9%)
	Stage III	179 (29.9%)
	Stage IV	88 (14.7%)
Gender (%)	Female	289 (46.7%)
	Male	330 (53.3%)
CEA level (%)	<=5	252 (63.5%)
	>5	145 (36.5%)
History of colon polyps (%)	No	364 (68.4%)
	Yes	168 (31.6%)
Colon polyps present (%)	No	207 (69.5%)
	Yes	91 (30.5%)
Neoplasm type (%)	Colon adenocarcinoma	454 (73.3%)
	Rectum adenocarcinoma	165 (26.7%)
TP53 status (%)	Mut	312 (59.3%)
	WT	214 (40.7%)
KRAS status (%)	Mut	214 (40.7%)
	WT	312 (59.3%)
PIK3CA status (%)	Mut	133 (25.3%)
	WT	393 (74.7%)
Residual tumor (%)	R0	450 (91.5%)
	R1	6 (1.2%)
	R2	36 (7.3%)
Race (%)	Asian	12 (3.3%)
	Black or African American	65 (17.6%)
	White	292 (79.1%)
Age (%)	<=65	269 (43.5%)
	>65	350 (56.5%)
Weight (%)	<=90	231 (71.5%)
	>90	92 (28.5%)
Height (%)	<170	146 (48.0%)
	> =170	158 (52.0%)
Age (median [IQR])		68.00 [58.00, 76.00]
Height (median [IQR])		170.00 [162.00, 176.22]
Weight (median [IQR])		79.60 [65.00, 92.50]

**Table 2:** Association between the expression of TMUB1 and clinicopathological features.

Characters	Level	Low expression of TMUB1	High expression of TMUB1	*P*	Test
*n*		310	309		
T stage (%)	T1	5 (1.6%)	15 (4.9%)	0.046	
	T2	46 (14.9%)	59 (19.1%)		
	T3	218 (70.8%)	204 (66.0%)		
	T4	39 (12.7%)	31 (10.0%)		
N stage (%)	N0	172 (56.0%)	179 (57.9%)	0.888	
	N1	76 (24.8%)	74 (23.9%)		
	N2	59 (19.2%)	56 (18.1%)		
M stage (%)	M0	226 (86.3%)	233 (82.0%)	0.219	
	M1	36 (13.7%)	51 (18.0%)		
Pathologic stage (%)	Stage I	43 (14.5%)	62 (20.5%)	0.065	Exact
	Stage II	121 (40.9%)	106 (35.0%)		
	Stage III	95 (32.1%)	84 (27.7%)		
	Stage IV	37 (12.5%)	51 (16.8%)		
Gender (%)	Female	148 (47.7%)	141 (45.6%)	0.656	
	Male	162 (52.3%)	168 (54.4%)		
CEA level (%)	<=5	124 (61.7%)	128 (65.3%)	0.52	
	>5	77 (38.3%)	68 (34.7%)		
History of colon polyps (%)	NO	182 (69.7%)	182 (67.2%)	0.586	
	YES	79 (30.3%)	89 (32.8%)		
Colon polyps present (%)	NO	135 (70.7%)	72 (67.3%)	0.632	
	YES	56 (29.3%)	35 (32.7%)		
Neoplasm type (%)	Colon adenocarcinoma	225 (72.6%)	229 (74.1%)	0.734	
	Rectum adenocarcinoma	85 (27.4%)	80 (25.9%)		
TP53 status (%)	Mut	154 (55.8%)	158 (63.2%)	0.102	
	WT	122 (44.2%)	92 (36.8%)		
KRAS status (%)	Mut	114 (41.3%)	100 (40.0%)	0.83	
	WT	162 (58.7%)	150 (60.0%)		
PIK3CA status (%)	Mut	72 (26.1%)	61 (24.4%)	0.731	
	WT	204 (73.9%)	189 (75.6%)		
Residual tumor (%)	R0	218 (94.8%)	232 (88.5%)	0.001	
	R1	5 (2.2%)	1 (0.4%)		
	R2	7 (3.0%)	29 (11.1%)		
Race (%)	Asian	8 (3.4%)	4 (3.0%)	0.21	
	Black or African American	35 (15.0%)	30 (22.2%)		
	White	191 (81.6%)	101 (74.8%)		
Age (%)	<=65	144 (46.5%)	125 (40.5%)	0.154	
	>65	166 (53.5%)	184 (59.5%)		
Weight (%)	<=90	147 (72.8%)	84 (69.4%)	0.604	
	>90	55 (27.2%)	37 (30.6%)		
Height (%)	<170	92 (46.9%)	54 (50.0%)	0.696	
	> =170	104 (53.1%)	54 (50.0%)		
Age (median [IQR])		67.00 [57.00, 75.00]	68.00 [59.00, 77.00]	0.165	Nonnorm
Height (median [IQR])		170.00 [162.00, 177.00]	169.00 [162.00, 175.25]	0.577	Nonnorm
Weight (median [IQR])		80.00 [65.43, 91.00]	78.90 [64.50, 94.00]	0.876	Nonnorm

**Table 3:** Expression of TMUB1 associated with clinicopathological characteristics (logistic regression).

Characteristics	Odds ratio in TMUB1 expression	Odds ratio (OR)	*P* value
T stage (T3 and T4 vs. T1 and T2)	617	0.63 (0.42-0.94)	0.023
N stage (N1 and N2 vs. N0)	616	0.93 (0.67-1.27)	0.633
M stage (M1 vs. M0)	546	1.37 (0.87-2.20)	0.18
Pathologic stage (stage III and stage IV vs. stage I and stage II)	599	1.00 (0.72-1.38)	0.992
Neoplasm type (rectum adenocarcinoma vs. colon adenocarcinoma)	619	0.92 (0.65-1.32)	0.667
Residual tumor (R1 and R2 vs. R0)	492	2.35 (1.20-4.88)	0.016
CEA level (>5 vs. <=5)	397	0.86 (0.57-1.29)	0.455
TP53 status (Mut vs. WT)	526	1.36 (0.96-1.93)	0.085
KRAS status (Mut vs. WT)	526	0.95 (0.67-1.34)	0.761
PIK3CA status (Mut vs. WT)	526	0.91 (0.62-1.36)	0.657

**Table 4:** Survival outcomes and multivariate analysis of TCGA data.

Characteristics	Total (*N*)	HR (95% CI)	*P* value
A			
T stage (T1 and T2 vs. T3 and T4)	616	0.416 (0.223-0.773)	0.006
N stage (N1 and N2 vs. N0)	615	2.567 (1.787-3.686)	<0.001
M stage (M1 vs. M0)	545	4.096 (2.752-6.096)	<0.001
Age (>65 vs. <=65)	618	2.023 (1.371-2.986)	<0.001
Weight (>90 vs. <=90)	323	0.756 (0.412-1.388)	0.367
Height (> =170 vs. <170)	304	0.773 (0.466-1.282)	0.318
Gender (male vs. female)	618	1.056 (0.744-1.498)	0.762
Race (White vs. Asian and Black or African American)	369	0.933 (0.541-1.610)	0.803
History of colon polyps (yes vs. no)	531	0.832 (0.522-1.326)	0.44
Colon polyps present (yes vs. no)	298	1.316 (0.777-2.229)	0.307
Residual tumor (R1 and R2 vs. R0)	491	4.466 (2.715-7.347)	<0.001
CEA level (>5 vs. <=5)	396	2.697 (1.646-4.418)	<0.001
TP53 status (Mut vs. WT)	526	1.119 (0.768-1.632)	0.558
KRAS status (Mut vs. WT)	526	0.954 (0.657-1.385)	0.805
PIK3CA status (Mut vs. WT)	526	0.892 (0.579-1.375)	0.605
Pathologic stage (stage I vs. stage III, stage IV, and stage II)	598	0.328 (0.153-0.705)	0.004
TMUB1 (high vs. low)	618	1.734 (1.216-2.473)	0.002
B			
T stage (T1 and T2 vs. T3 and T4)	616	0.530 (0.067-4.195)	0.548
N stage (N1 and N2 vs. N0)	615	1.362 (0.605-3.065)	0.455
M stage (M1 vs. M0)	545	2.212 (0.846-5.781)	0.105
Age (>65 vs. <=65)	618	2.708 (1.306-5.617)	0.007
Residual tumor (R1 and R2 vs. R0)	491	2.001 (0.880-4.547)	0.098
CEA level (>5 vs. <=5)	396	1.574 (0.802-3.086)	0.187
Pathologic stage (stage I vs. stage III, stage IV, and stage II)	598	0.351 (0.020-6.291)	0.477
TMUB1 (high vs. low)	618	1.960 (1.042-3.686)	0.037

(A) Association with overall survival and clinicopathological characteristics in patients with colorectal cancer using Cox regression. (B) Multivariate survival model after selection of variables.

**Table 5:** Disease-specific survival and multivariate analysis of TCGA data.

Characteristics	Total (*N*)	HR (95% CI)	*P* value
A			
T stage (T1 and T2 vs. T3 and T4)	594	0.157 (0.050-0.500)	0.002
N stage (N1 and N2 vs. N0)	593	4.065 (2.463-6.710)	<0.001
M stage (M1 vs. M0)	524	7.531 (4.683-12.111)	<0.001
Age (>65 vs. <=65)	596	1.468 (0.924-2.332)	0.104
Weight (>90 vs. <=90)	302	1.067 (0.492-2.314)	0.87
Height (> =170 vs. <170)	284	0.821 (0.388-1.734)	0.604
Gender (male vs. female)	596	1.206 (0.768-1.893)	0.415
Race (White vs. Asian and Black or African American)	347	0.493 (0.254-0.959)	0.037
History of colon polyps (yes vs. no)	518	0.988 (0.574-1.698)	0.964
Colon polyps present (yes vs. no)	290	1.386 (0.683-2.811)	0.365
Residual tumor (R1 and R2 vs. R0)	490	6.140 (3.607-10.453)	<0.001
CEA level (>5 vs. <=5)	395	2.889 (1.610-5.187)	<0.001
TP53 status (Mut vs. WT)	504	0.967 (0.595-1.571)	0.891
KRAS status (Mut vs. WT)	504	1.196 (0.739-1.935)	0.466
PIK3CA status (Mut vs. WT)	504	0.928 (0.529-1.629)	0.795
Pathologic stage (stage I vs. stage III, stage IV, and stage II)	577	0.132 (0.032-0.537)	0.005
TMUB1 (high vs. low)	596	2.003 (1.264-3.174)	0.003
B			
T stage (T1 and T2 vs. T3 and T4)	594	0.789 (0.098-6.374)	0.824
N stage (N1 and N2 vs. N0)	593	0.833 (0.324-2.140)	0.704
M stage (M1 vs. M0)	524	4.540 (1.574-13.096)	0.005
Residual tumor (R1 and R2 vs. R0)	490	1.727 (0.729-4.093)	0.214
CEA level (>5 vs. <=5)	395	1.676 (0.786-3.574)	0.181
Pathologic stage (stage I vs. stage III, stage IV, and Stage II)	577	0.000 (0.000-Inf)	0.997
TMUB1 (high vs. low)	596	2.538 (1.204-5.350)	0.014

(A) Association with disease-specific survival and clinicopathological characteristics in patients with colorectal cancer using Cox regression. (B) Multivariate survival model after selection of variables.

## Data Availability

The data used to support the findings of this study are included within the article.
